# Supplementary material for: Pyruvate Dehydrogenase Kinase 1 inhibition mediated oxidative phosphorylation enhancement in cartilage promotes osteoarthritis progression
Source: BMC Musculoskelet Disord. 2023 Jul 20;24:597. doi: 10.1186/s12891-023-06585-6 (PMC10357736; doi:10.1186/s12891-023-06585-6)
Supplement: Supplementary file 1 — Additional file 1. [file 12891_2023_6585_MOESM1_ESM.zip › BP.Gsea.1653623667859/GOBP_SULFUR_COMPOUND_METABOLIC_PROCESS.html]

Details for gene set GOBP\_SULFUR\_COMPOUND\_METABOLIC\_PROCESS[GSEA]

|  || Dataset | 55457\_collapsed\_to\_symbols.cls.cls #OA\_versus\_normal.cls.cls #OA\_versus\_normal\_repos |
| Phenotype | cls.cls#OA\_versus\_normal\_repos |
| Upregulated in class | OA |
| GeneSet | GOBP\_SULFUR\_COMPOUND\_METABOLIC\_PROCESS |
| Enrichment Score (ES) | 0.27747732 |
| Normalized Enrichment Score (NES) | 1.4813315 |
| Nominal p-value | 0.0 |
| FDR q-value | 0.38315833 |
| FWER p-Value | 1.0 |
Table: GSEA Results Summary

  

Fig 1: Enrichment plot: GOBP\_SULFUR\_COMPOUND\_METABOLIC\_PROCESS      
 Profile of the Running ES Score & Positions of GeneSet Members on the Rank Ordered List

  

| SYMBOL | TITLE | RANK IN GENE LIST | RANK METRIC SCORE | RUNNING ES | CORE ENRICHMENT || 1 | MPC2 | mitochondrial pyruvate carrier 2 [Source:HGNC Symbol;Acc:HGNC:24515] | 140 | 0.870 | -0.0006 | Yes |
| 2 | PDHB | pyruvate dehydrogenase E1 subunit beta [Source:HGNC Symbol;Acc:HGNC:8808] | 149 | 0.862 | 0.0092 | Yes |
| 3 | EXT2 | exostosin glycosyltransferase 2 [Source:HGNC Symbol;Acc:HGNC:3513] | 170 | 0.838 | 0.0178 | Yes |
| 4 | GSTA4 | glutathione S-transferase alpha 4 [Source:HGNC Symbol;Acc:HGNC:4629] | 216 | 0.808 | 0.0240 | Yes |
| 5 | GLRX5 | glutaredoxin 5 [Source:HGNC Symbol;Acc:HGNC:20134] | 263 | 0.778 | 0.0298 | Yes |
| 6 | B4GAT1 | "beta-1,4-glucuronyltransferase 1 [Source:HGNC Symbol;Acc:HGNC:15685]" | 282 | 0.764 | 0.0376 | Yes |
| 7 | GLB1 | galactosidase beta 1 [Source:HGNC Symbol;Acc:HGNC:4298] | 337 | 0.731 | 0.0421 | Yes |
| 8 | PMVK | phosphomevalonate kinase [Source:HGNC Symbol;Acc:HGNC:9141] | 346 | 0.725 | 0.0503 | Yes |
| 9 | MSRA | methionine sulfoxide reductase A [Source:HGNC Symbol;Acc:HGNC:7377] | 380 | 0.715 | 0.0563 | Yes |
| 10 | THTPA | thiamine triphosphatase [Source:HGNC Symbol;Acc:HGNC:18987] | 418 | 0.698 | 0.0618 | Yes |
| 11 | GSTK1 | glutathione S-transferase kappa 1 [Source:HGNC Symbol;Acc:HGNC:16906] | 490 | 0.662 | 0.0642 | Yes |
| 12 | GSS | glutathione synthetase [Source:HGNC Symbol;Acc:HGNC:4624] | 502 | 0.658 | 0.0713 | Yes |
| 13 | TPK1 | thiamin pyrophosphokinase 1 [Source:HGNC Symbol;Acc:HGNC:17358] | 511 | 0.656 | 0.0786 | Yes |
| 14 | DLD | dihydrolipoamide dehydrogenase [Source:HGNC Symbol;Acc:HGNC:2898] | 565 | 0.635 | 0.0821 | Yes |
| 15 | ETHE1 | ETHE1 persulfide dioxygenase [Source:HGNC Symbol;Acc:HGNC:23287] | 572 | 0.632 | 0.0892 | Yes |
| 16 | CLIC3 | chloride intracellular channel 3 [Source:HGNC Symbol;Acc:HGNC:2064] | 582 | 0.629 | 0.0961 | Yes |
| 17 | PAPSS2 | 3'-phosphoadenosine 5'-phosphosulfate synthase 2 [Source:HGNC Symbol;Acc:HGNC:8604] | 592 | 0.627 | 0.1030 | Yes |
| 18 | HPGDS | hematopoietic prostaglandin D synthase [Source:HGNC Symbol;Acc:HGNC:17890] | 605 | 0.622 | 0.1096 | Yes |
| 19 | BTD | biotinidase [Source:HGNC Symbol;Acc:HGNC:1122] | 613 | 0.619 | 0.1165 | Yes |
| 20 | MMACHC | metabolism of cobalamin associated C [Source:HGNC Symbol;Acc:HGNC:24525] | 642 | 0.608 | 0.1216 | Yes |
| 21 | APIP | APAF1 interacting protein [Source:HGNC Symbol;Acc:HGNC:17581] | 643 | 0.607 | 0.1290 | Yes |
| 22 | PAPSS1 | 3'-phosphoadenosine 5'-phosphosulfate synthase 1 [Source:HGNC Symbol;Acc:HGNC:8603] | 703 | 0.593 | 0.1315 | Yes |
| 23 | MAT2B | methionine adenosyltransferase 2B [Source:HGNC Symbol;Acc:HGNC:6905] | 715 | 0.589 | 0.1377 | Yes |
| 24 | PPCS | phosphopantothenoylcysteine synthetase [Source:HGNC Symbol;Acc:HGNC:25686] | 716 | 0.589 | 0.1449 | Yes |
| 25 | HSD17B12 | hydroxysteroid 17-beta dehydrogenase 12 [Source:HGNC Symbol;Acc:HGNC:18646] | 795 | 0.570 | 0.1456 | Yes |
| 26 | AKR1A1 | aldo-keto reductase family 1 member A1 [Source:HGNC Symbol;Acc:HGNC:380] | 808 | 0.567 | 0.1515 | Yes |
| 27 | ACOT7 | acyl-CoA thioesterase 7 [Source:HGNC Symbol;Acc:HGNC:24157] | 971 | 0.524 | 0.1450 | Yes |
| 28 | GSTZ1 | glutathione S-transferase zeta 1 [Source:HGNC Symbol;Acc:HGNC:4643] | 974 | 0.523 | 0.1512 | Yes |
| 29 | CHST12 | carbohydrate sulfotransferase 12 [Source:HGNC Symbol;Acc:HGNC:17423] | 985 | 0.521 | 0.1567 | Yes |
| 30 | HYAL1 | hyaluronidase 1 [Source:HGNC Symbol;Acc:HGNC:5320] | 997 | 0.518 | 0.1621 | Yes |
| 31 | ACAT1 | acetyl-CoA acetyltransferase 1 [Source:HGNC Symbol;Acc:HGNC:93] | 1017 | 0.513 | 0.1668 | Yes |
| 32 | GSTM3 | glutathione S-transferase mu 3 [Source:HGNC Symbol;Acc:HGNC:4635] | 1025 | 0.512 | 0.1724 | Yes |
| 33 | PPT1 | palmitoyl-protein thioesterase 1 [Source:HGNC Symbol;Acc:HGNC:9325] | 1042 | 0.508 | 0.1773 | Yes |
| 34 | TECR | "trans-2,3-enoyl-CoA reductase [Source:HGNC Symbol;Acc:HGNC:4551]" | 1047 | 0.508 | 0.1831 | Yes |
| 35 | ACOT8 | acyl-CoA thioesterase 8 [Source:HGNC Symbol;Acc:HGNC:15919] | 1088 | 0.500 | 0.1860 | Yes |
| 36 | GSTT1 | glutathione S-transferase theta 1 [Source:HGNC Symbol;Acc:HGNC:4641] | 1129 | 0.491 | 0.1888 | Yes |
| 37 | ICMT | isoprenylcysteine carboxyl methyltransferase [Source:HGNC Symbol;Acc:HGNC:5350] | 1140 | 0.488 | 0.1939 | Yes |
| 38 | TPST2 | tyrosylprotein sulfotransferase 2 [Source:HGNC Symbol;Acc:HGNC:12021] | 1197 | 0.477 | 0.1952 | Yes |
| 39 | CLIC2 | chloride intracellular channel 2 [Source:HGNC Symbol;Acc:HGNC:2063] | 1214 | 0.473 | 0.1997 | Yes |
| 40 | GSTP1 | glutathione S-transferase pi 1 [Source:HGNC Symbol;Acc:HGNC:4638] | 1260 | 0.465 | 0.2017 | Yes |
| 41 | OXSM | "3-oxoacyl-ACP synthase, mitochondrial [Source:HGNC Symbol;Acc:HGNC:26063]" | 1342 | 0.451 | 0.2008 | Yes |
| 42 | HSD17B4 | hydroxysteroid 17-beta dehydrogenase 4 [Source:HGNC Symbol;Acc:HGNC:5213] | 1354 | 0.450 | 0.2053 | Yes |
| 43 | HAGH | hydroxyacylglutathione hydrolase [Source:HGNC Symbol;Acc:HGNC:4805] | 1395 | 0.443 | 0.2075 | Yes |
| 44 | PARK7 | Parkinsonism associated deglycase [Source:HGNC Symbol;Acc:HGNC:16369] | 1406 | 0.442 | 0.2121 | Yes |
| 45 | B3GNT2 | "UDP-GlcNAc:betaGal beta-1,3-N-acetylglucosaminyltransferase 2 [Source:HGNC Symbol;Acc:HGNC:15629]" | 1427 | 0.439 | 0.2158 | Yes |
| 46 | ANGPT1 | angiopoietin 1 [Source:HGNC Symbol;Acc:HGNC:484] | 1467 | 0.433 | 0.2180 | Yes |
| 47 | SUCLG2 | succinate-CoA ligase GDP-forming subunit beta [Source:HGNC Symbol;Acc:HGNC:11450] | 1472 | 0.432 | 0.2229 | Yes |
| 48 | CIAO2B | cytosolic iron-sulfur assembly component 2B [Source:HGNC Symbol;Acc:HGNC:24261] | 1509 | 0.428 | 0.2252 | Yes |
| 49 | ISCU | iron-sulfur cluster assembly enzyme [Source:HGNC Symbol;Acc:HGNC:29882] | 1530 | 0.423 | 0.2287 | Yes |
| 50 | NOX4 | NADPH oxidase 4 [Source:HGNC Symbol;Acc:HGNC:7891] | 1563 | 0.418 | 0.2313 | Yes |
| 51 | NDST2 | N-deacetylase and N-sulfotransferase 2 [Source:HGNC Symbol;Acc:HGNC:7681] | 1587 | 0.415 | 0.2345 | Yes |
| 52 | NFS1 | NFS1 cysteine desulfurase [Source:HGNC Symbol;Acc:HGNC:15910] | 1741 | 0.393 | 0.2271 | Yes |
| 53 | PDK2 | pyruvate dehydrogenase kinase 2 [Source:HGNC Symbol;Acc:HGNC:8810] | 1772 | 0.388 | 0.2294 | Yes |
| 54 | TDO2 | "tryptophan 2,3-dioxygenase [Source:HGNC Symbol;Acc:HGNC:11708]" | 1783 | 0.387 | 0.2333 | Yes |
| 55 | SNCA | synuclein alpha [Source:HGNC Symbol;Acc:HGNC:11138] | 1800 | 0.384 | 0.2367 | Yes |
| 56 | ACSM3 | acyl-CoA synthetase medium chain family member 3 [Source:HGNC Symbol;Acc:HGNC:10522] | 1835 | 0.380 | 0.2386 | Yes |
| 57 | HMGCS2 | 3-hydroxy-3-methylglutaryl-CoA synthase 2 [Source:HGNC Symbol;Acc:HGNC:5008] | 1888 | 0.374 | 0.2390 | Yes |
| 58 | XYLT2 | xylosyltransferase 2 [Source:HGNC Symbol;Acc:HGNC:15517] | 1938 | 0.368 | 0.2396 | Yes |
| 59 | ADI1 | acireductone dioxygenase 1 [Source:HGNC Symbol;Acc:HGNC:30576] | 2004 | 0.360 | 0.2388 | Yes |
| 60 | ELOVL4 | ELOVL fatty acid elongase 4 [Source:HGNC Symbol;Acc:HGNC:14415] | 2017 | 0.360 | 0.2422 | Yes |
| 61 | NFE2L2 | "nuclear factor, erythroid 2 like 2 [Source:HGNC Symbol;Acc:HGNC:7782]" | 2034 | 0.357 | 0.2453 | Yes |
| 62 | PDK1 | pyruvate dehydrogenase kinase 1 [Source:HGNC Symbol;Acc:HGNC:8809] | 2124 | 0.347 | 0.2424 | Yes |
| 63 | GLO1 | glyoxalase I [Source:HGNC Symbol;Acc:HGNC:4323] | 2141 | 0.345 | 0.2453 | Yes |
| 64 | CPS1 | carbamoyl-phosphate synthase 1 [Source:HGNC Symbol;Acc:HGNC:2323] | 2178 | 0.341 | 0.2466 | Yes |
| 65 | SQOR | sulfide quinone oxidoreductase [Source:HGNC Symbol;Acc:HGNC:20390] | 2225 | 0.336 | 0.2470 | Yes |
| 66 | CHPF2 | chondroitin polymerizing factor 2 [Source:HGNC Symbol;Acc:HGNC:29270] | 2281 | 0.329 | 0.2466 | Yes |
| 67 | LYRM4 | LYR motif containing 4 [Source:HGNC Symbol;Acc:HGNC:21365] | 2338 | 0.322 | 0.2461 | Yes |
| 68 | HPSE | heparanase [Source:HGNC Symbol;Acc:HGNC:5164] | 2360 | 0.319 | 0.2483 | Yes |
| 69 | SULT1A1 | sulfotransferase family 1A member 1 [Source:HGNC Symbol;Acc:HGNC:11453] | 2410 | 0.313 | 0.2482 | Yes |
| 70 | SOD1 | superoxide dismutase 1 [Source:HGNC Symbol;Acc:HGNC:11179] | 2413 | 0.313 | 0.2518 | Yes |
| 71 | PDHA1 | pyruvate dehydrogenase E1 subunit alpha 1 [Source:HGNC Symbol;Acc:HGNC:8806] | 2433 | 0.310 | 0.2541 | Yes |
| 72 | CSGALNACT1 | chondroitin sulfate N-acetylgalactosaminyltransferase 1 [Source:HGNC Symbol;Acc:HGNC:24290] | 2507 | 0.302 | 0.2520 | Yes |
| 73 | TPST1 | tyrosylprotein sulfotransferase 1 [Source:HGNC Symbol;Acc:HGNC:12020] | 2512 | 0.302 | 0.2553 | Yes |
| 74 | ACSBG1 | acyl-CoA synthetase bubblegum family member 1 [Source:HGNC Symbol;Acc:HGNC:29567] | 2520 | 0.301 | 0.2584 | Yes |
| 75 | TKTL1 | transketolase like 1 [Source:HGNC Symbol;Acc:HGNC:11835] | 2528 | 0.300 | 0.2615 | Yes |
| 76 | ARL6IP5 | ADP ribosylation factor like GTPase 6 interacting protein 5 [Source:HGNC Symbol;Acc:HGNC:16937] | 2545 | 0.298 | 0.2638 | Yes |
| 77 | NUBP1 | nucleotide binding protein 1 [Source:HGNC Symbol;Acc:HGNC:8041] | 2568 | 0.295 | 0.2656 | Yes |
| 78 | NUBPL | nucleotide binding protein like [Source:HGNC Symbol;Acc:HGNC:20278] | 2569 | 0.295 | 0.2692 | Yes |
| 79 | MGST2 | microsomal glutathione S-transferase 2 [Source:HGNC Symbol;Acc:HGNC:7063] | 2583 | 0.292 | 0.2717 | Yes |
| 80 | CDO1 | cysteine dioxygenase type 1 [Source:HGNC Symbol;Acc:HGNC:1795] | 2635 | 0.287 | 0.2711 | Yes |
| 81 | SULT1E1 | sulfotransferase family 1E member 1 [Source:HGNC Symbol;Acc:HGNC:11377] | 2791 | 0.271 | 0.2621 | Yes |
| 82 | TST | thiosulfate sulfurtransferase [Source:HGNC Symbol;Acc:HGNC:12388] | 2808 | 0.269 | 0.2641 | Yes |
| 83 | SULT1A2 | sulfotransferase family 1A member 2 [Source:HGNC Symbol;Acc:HGNC:11454] | 2881 | 0.262 | 0.2616 | Yes |
| 84 | LIAS | lipoic acid synthetase [Source:HGNC Symbol;Acc:HGNC:16429] | 2917 | 0.259 | 0.2619 | Yes |
| 85 | GAL3ST4 | galactose-3-O-sulfotransferase 4 [Source:HGNC Symbol;Acc:HGNC:24145] | 2979 | 0.252 | 0.2602 | Yes |
| 86 | NDNF | neuron derived neurotrophic factor [Source:HGNC Symbol;Acc:HGNC:26256] | 2988 | 0.251 | 0.2626 | Yes |
| 87 | FAR2 | fatty acyl-CoA reductase 2 [Source:HGNC Symbol;Acc:HGNC:25531] | 2999 | 0.250 | 0.2648 | Yes |
| 88 | SPOCK3 | "SPARC (osteonectin), cwcv and kazal like domains proteoglycan 3 [Source:HGNC Symbol;Acc:HGNC:13565]" | 3046 | 0.245 | 0.2641 | Yes |
| 89 | NFU1 | NFU1 iron-sulfur cluster scaffold [Source:HGNC Symbol;Acc:HGNC:16287] | 3086 | 0.241 | 0.2639 | Yes |
| 90 | AHCYL2 | adenosylhomocysteinase like 2 [Source:HGNC Symbol;Acc:HGNC:22204] | 3099 | 0.240 | 0.2659 | Yes |
| 91 | GSTM5 | glutathione S-transferase mu 5 [Source:HGNC Symbol;Acc:HGNC:4637] | 3123 | 0.238 | 0.2670 | Yes |
| 92 | GSTM4 | glutathione S-transferase mu 4 [Source:HGNC Symbol;Acc:HGNC:4636] | 3144 | 0.235 | 0.2682 | Yes |
| 93 | UST | uronyl 2-sulfotransferase [Source:HGNC Symbol;Acc:HGNC:17223] | 3180 | 0.233 | 0.2683 | Yes |
| 94 | SUCLA2 | succinate-CoA ligase ADP-forming subunit beta [Source:HGNC Symbol;Acc:HGNC:11448] | 3228 | 0.228 | 0.2673 | Yes |
| 95 | ELOVL6 | ELOVL fatty acid elongase 6 [Source:HGNC Symbol;Acc:HGNC:15829] | 3253 | 0.226 | 0.2681 | Yes |
| 96 | EXT1 | exostosin glycosyltransferase 1 [Source:HGNC Symbol;Acc:HGNC:3512] | 3321 | 0.219 | 0.2655 | Yes |
| 97 | MTAP | methylthioadenosine phosphorylase [Source:HGNC Symbol;Acc:HGNC:7413] | 3324 | 0.219 | 0.2679 | Yes |
| 98 | GLRX3 | glutaredoxin 3 [Source:HGNC Symbol;Acc:HGNC:15987] | 3336 | 0.218 | 0.2697 | Yes |
| 99 | CTNS | "cystinosin, lysosomal cystine transporter [Source:HGNC Symbol;Acc:HGNC:2518]" | 3337 | 0.218 | 0.2723 | Yes |
| 100 | ST3GAL6 | "ST3 beta-galactoside alpha-2,3-sialyltransferase 6 [Source:HGNC Symbol;Acc:HGNC:18080]" | 3339 | 0.218 | 0.2749 | Yes |
| 101 | MMUT | methylmalonyl-CoA mutase [Source:HGNC Symbol;Acc:HGNC:7526] | 3362 | 0.215 | 0.2758 | Yes |
| 102 | ENPP1 | ectonucleotide pyrophosphatase/phosphodiesterase 1 [Source:HGNC Symbol;Acc:HGNC:3356] | 3392 | 0.212 | 0.2760 | Yes |
| 103 | B4GALT4 | "beta-1,4-galactosyltransferase 4 [Source:HGNC Symbol;Acc:HGNC:927]" | 3407 | 0.211 | 0.2775 | Yes |
| 104 | NDST4 | N-deacetylase and N-sulfotransferase 4 [Source:HGNC Symbol;Acc:HGNC:20779] | 3497 | 0.202 | 0.2729 | No |
| 105 | BGN | biglycan [Source:HGNC Symbol;Acc:HGNC:1044] | 3564 | 0.197 | 0.2700 | No |
| 106 | PAX8 | paired box 8 [Source:HGNC Symbol;Acc:HGNC:8622] | 3680 | 0.187 | 0.2632 | No |
| 107 | CIAO3 | cytosolic iron-sulfur assembly component 3 [Source:HGNC Symbol;Acc:HGNC:14179] | 3784 | 0.178 | 0.2572 | No |
| 108 | SUOX | sulfite oxidase [Source:HGNC Symbol;Acc:HGNC:11460] | 3801 | 0.177 | 0.2581 | No |
| 109 | GSTA3 | glutathione S-transferase alpha 3 [Source:HGNC Symbol;Acc:HGNC:4628] | 3807 | 0.176 | 0.2598 | No |
| 110 | GLYAT | glycine-N-acyltransferase [Source:HGNC Symbol;Acc:HGNC:13734] | 3871 | 0.167 | 0.2568 | No |
| 111 | XYLT1 | xylosyltransferase 1 [Source:HGNC Symbol;Acc:HGNC:15516] | 3916 | 0.163 | 0.2553 | No |
| 112 | SULT1A3 | sulfotransferase family 1A member 3 [Source:HGNC Symbol;Acc:HGNC:11455] | 3923 | 0.162 | 0.2568 | No |
| 113 | DGAT1 | diacylglycerol O-acyltransferase 1 [Source:HGNC Symbol;Acc:HGNC:2843] | 3950 | 0.160 | 0.2567 | No |
| 114 | BOLA2 | bolA family member 2 [Source:HGNC Symbol;Acc:HGNC:29488] | 4010 | 0.155 | 0.2539 | No |
| 115 | ACOT2 | acyl-CoA thioesterase 2 [Source:HGNC Symbol;Acc:HGNC:18431] | 4046 | 0.152 | 0.2530 | No |
| 116 | ACSM2A | acyl-CoA synthetase medium chain family member 2A [Source:HGNC Symbol;Acc:HGNC:32017] | 4080 | 0.148 | 0.2522 | No |
| 117 | ISCA1 | iron-sulfur cluster assembly 1 [Source:HGNC Symbol;Acc:HGNC:28660] | 4116 | 0.144 | 0.2511 | No |
| 118 | CIAPIN1 | cytokine induced apoptosis inhibitor 1 [Source:HGNC Symbol;Acc:HGNC:28050] | 4161 | 0.141 | 0.2494 | No |
| 119 | MTHFR | methylenetetrahydrofolate reductase [Source:HGNC Symbol;Acc:HGNC:7436] | 4334 | 0.125 | 0.2373 | No |
| 120 | SPOCK2 | "SPARC (osteonectin), cwcv and kazal like domains proteoglycan 2 [Source:HGNC Symbol;Acc:HGNC:13564]" | 4592 | 0.105 | 0.2182 | No |
| 121 | GSR | glutathione-disulfide reductase [Source:HGNC Symbol;Acc:HGNC:4623] | 4597 | 0.105 | 0.2191 | No |
| 122 | EDNRA | endothelin receptor type A [Source:HGNC Symbol;Acc:HGNC:3179] | 4663 | 0.099 | 0.2152 | No |
| 123 | CHST11 | carbohydrate sulfotransferase 11 [Source:HGNC Symbol;Acc:HGNC:17422] | 4664 | 0.099 | 0.2164 | No |
| 124 | ABCB7 | ATP binding cassette subfamily B member 7 [Source:HGNC Symbol;Acc:HGNC:48] | 4669 | 0.099 | 0.2173 | No |
| 125 | HS3ST3B1 | heparan sulfate-glucosamine 3-sulfotransferase 3B1 [Source:HGNC Symbol;Acc:HGNC:5198] | 4683 | 0.098 | 0.2174 | No |
| 126 | CLIC1 | chloride intracellular channel 1 [Source:HGNC Symbol;Acc:HGNC:2062] | 4704 | 0.095 | 0.2170 | No |
| 127 | MLYCD | malonyl-CoA decarboxylase [Source:HGNC Symbol;Acc:HGNC:7150] | 4852 | 0.083 | 0.2064 | No |
| 128 | ACSM5 | acyl-CoA synthetase medium chain family member 5 [Source:HGNC Symbol;Acc:HGNC:26060] | 4976 | 0.074 | 0.1975 | No |
| 129 | IDUA | alpha-L-iduronidase [Source:HGNC Symbol;Acc:HGNC:5391] | 5002 | 0.073 | 0.1964 | No |
| 130 | CHST3 | carbohydrate sulfotransferase 3 [Source:HGNC Symbol;Acc:HGNC:1971] | 5169 | 0.058 | 0.1840 | No |
| 131 | GGT1 | gamma-glutamyltransferase 1 [Source:HGNC Symbol;Acc:HGNC:4250] | 5177 | 0.058 | 0.1842 | No |
| 132 | CBS | cystathionine beta-synthase [Source:HGNC Symbol;Acc:HGNC:1550] | 5285 | 0.049 | 0.1763 | No |
| 133 | AHCY | adenosylhomocysteinase [Source:HGNC Symbol;Acc:HGNC:343] | 5307 | 0.047 | 0.1752 | No |
| 134 | OPLAH | "5-oxoprolinase, ATP-hydrolysing [Source:HGNC Symbol;Acc:HGNC:8149]" | 5339 | 0.045 | 0.1733 | No |
| 135 | HACD1 | 3-hydroxyacyl-CoA dehydratase 1 [Source:HGNC Symbol;Acc:HGNC:9639] | 5342 | 0.044 | 0.1736 | No |
| 136 | NDST3 | N-deacetylase and N-sulfotransferase 3 [Source:HGNC Symbol;Acc:HGNC:7682] | 5446 | 0.037 | 0.1659 | No |
| 137 | ACOT9 | acyl-CoA thioesterase 9 [Source:HGNC Symbol;Acc:HGNC:17152] | 5451 | 0.037 | 0.1661 | No |
| 138 | STAT5B | signal transducer and activator of transcription 5B [Source:HGNC Symbol;Acc:HGNC:11367] | 5554 | 0.028 | 0.1583 | No |
| 139 | ENOPH1 | enolase-phosphatase 1 [Source:HGNC Symbol;Acc:HGNC:24599] | 5615 | 0.023 | 0.1539 | No |
| 140 | DLAT | dihydrolipoamide S-acetyltransferase [Source:HGNC Symbol;Acc:HGNC:2896] | 5647 | 0.020 | 0.1516 | No |
| 141 | SULT4A1 | sulfotransferase family 4A member 1 [Source:HGNC Symbol;Acc:HGNC:14903] | 5676 | 0.017 | 0.1496 | No |
| 142 | AHCYL1 | adenosylhomocysteinase like 1 [Source:HGNC Symbol;Acc:HGNC:344] | 5837 | 0.006 | 0.1371 | No |
| 143 | GSTM1 | glutathione S-transferase mu 1 [Source:HGNC Symbol;Acc:HGNC:4632] | 5848 | 0.005 | 0.1363 | No |
| 144 | ACSBG2 | acyl-CoA synthetase bubblegum family member 2 [Source:HGNC Symbol;Acc:HGNC:24174] | 5970 | -0.002 | 0.1268 | No |
| 145 | GLRX2 | glutaredoxin 2 [Source:HGNC Symbol;Acc:HGNC:16065] | 6116 | -0.014 | 0.1155 | No |
| 146 | BPNT2 | "3'(2'), 5'-bisphosphate nucleotidase 2 [Source:HGNC Symbol;Acc:HGNC:26019]" | 6136 | -0.015 | 0.1141 | No |
| 147 | GLCE | glucuronic acid epimerase [Source:HGNC Symbol;Acc:HGNC:17855] | 6148 | -0.016 | 0.1135 | No |
| 148 | HACD2 | 3-hydroxyacyl-CoA dehydratase 2 [Source:HGNC Symbol;Acc:HGNC:9640] | 6162 | -0.017 | 0.1126 | No |
| 149 | BHMT2 | betaine--homocysteine S-methyltransferase 2 [Source:HGNC Symbol;Acc:HGNC:1048] | 6165 | -0.018 | 0.1127 | No |
| 150 | PTGES2 | prostaglandin E synthase 2 [Source:HGNC Symbol;Acc:HGNC:17822] | 6269 | -0.025 | 0.1049 | No |
| 151 | ACP3 | acid phosphatase 3 [Source:HGNC Symbol;Acc:HGNC:125] | 6271 | -0.025 | 0.1051 | No |
| 152 | GSTO1 | glutathione S-transferase omega 1 [Source:HGNC Symbol;Acc:HGNC:13312] | 6275 | -0.025 | 0.1052 | No |
| 153 | SLC7A11 | solute carrier family 7 member 11 [Source:HGNC Symbol;Acc:HGNC:11059] | 6470 | -0.040 | 0.0903 | No |
| 154 | CHST7 | carbohydrate sulfotransferase 7 [Source:HGNC Symbol;Acc:HGNC:13817] | 6486 | -0.041 | 0.0896 | No |
| 155 | ACSL6 | acyl-CoA synthetase long chain family member 6 [Source:HGNC Symbol;Acc:HGNC:16496] | 6505 | -0.043 | 0.0887 | No |
| 156 | SLC25A1 | solute carrier family 25 member 1 [Source:HGNC Symbol;Acc:HGNC:10979] | 6509 | -0.043 | 0.0890 | No |
| 157 | MMS19 | "MMS19 homolog, cytosolic iron-sulfur assembly component [Source:HGNC Symbol;Acc:HGNC:13824]" | 6733 | -0.058 | 0.0720 | No |
| 158 | GHR | growth hormone receptor [Source:HGNC Symbol;Acc:HGNC:4263] | 6740 | -0.058 | 0.0723 | No |
| 159 | HLCS | holocarboxylase synthetase [Source:HGNC Symbol;Acc:HGNC:4976] | 6759 | -0.060 | 0.0715 | No |
| 160 | PDHX | pyruvate dehydrogenase complex component X [Source:HGNC Symbol;Acc:HGNC:21350] | 6760 | -0.060 | 0.0723 | No |
| 161 | CHST2 | carbohydrate sulfotransferase 2 [Source:HGNC Symbol;Acc:HGNC:1970] | 6778 | -0.061 | 0.0717 | No |
| 162 | PIPOX | pipecolic acid and sarcosine oxidase [Source:HGNC Symbol;Acc:HGNC:17804] | 6781 | -0.061 | 0.0723 | No |
| 163 | GGTLC1 | gamma-glutamyltransferase light chain 1 [Source:HGNC Symbol;Acc:HGNC:16437] | 6916 | -0.073 | 0.0625 | No |
| 164 | STAT5A | signal transducer and activator of transcription 5A [Source:HGNC Symbol;Acc:HGNC:11366] | 6988 | -0.079 | 0.0579 | No |
| 165 | SLC1A2 | solute carrier family 1 member 2 [Source:HGNC Symbol;Acc:HGNC:10940] | 7026 | -0.082 | 0.0559 | No |
| 166 | G6PD | glucose-6-phosphate dehydrogenase [Source:HGNC Symbol;Acc:HGNC:4057] | 7082 | -0.086 | 0.0526 | No |
| 167 | IDH1 | isocitrate dehydrogenase (NADP(+)) 1 [Source:HGNC Symbol;Acc:HGNC:5382] | 7109 | -0.089 | 0.0516 | No |
| 168 | GPX4 | glutathione peroxidase 4 [Source:HGNC Symbol;Acc:HGNC:4556] | 7115 | -0.089 | 0.0523 | No |
| 169 | GDAP1 | ganglioside induced differentiation associated protein 1 [Source:HGNC Symbol;Acc:HGNC:15968] | 7172 | -0.093 | 0.0490 | No |
| 170 | BHMT | betaine--homocysteine S-methyltransferase [Source:HGNC Symbol;Acc:HGNC:1047] | 7250 | -0.099 | 0.0441 | No |
| 171 | MICAL2 | "microtubule associated monooxygenase, calponin and LIM domain containing 2 [Source:HGNC Symbol;Acc:HGNC:24693]" | 7378 | -0.109 | 0.0354 | No |
| 172 | MCCC1 | methylcrotonyl-CoA carboxylase subunit 1 [Source:HGNC Symbol;Acc:HGNC:6936] | 7401 | -0.111 | 0.0350 | No |
| 173 | DSE | dermatan sulfate epimerase [Source:HGNC Symbol;Acc:HGNC:21144] | 7493 | -0.118 | 0.0292 | No |
| 174 | SLC26A1 | solute carrier family 26 member 1 [Source:HGNC Symbol;Acc:HGNC:10993] | 7618 | -0.128 | 0.0209 | No |
| 175 | PDK3 | pyruvate dehydrogenase kinase 3 [Source:HGNC Symbol;Acc:HGNC:8811] | 7661 | -0.132 | 0.0192 | No |
| 176 | GSTT2 | glutathione S-transferase theta 2 (gene/pseudogene) [Source:HGNC Symbol;Acc:HGNC:4642] | 7666 | -0.132 | 0.0205 | No |
| 177 | ACOT11 | acyl-CoA thioesterase 11 [Source:HGNC Symbol;Acc:HGNC:18156] | 7746 | -0.137 | 0.0159 | No |
| 178 | MTHFD2L | methylenetetrahydrofolate dehydrogenase (NADP+ dependent) 2 like [Source:HGNC Symbol;Acc:HGNC:31865] | 7789 | -0.141 | 0.0143 | No |
| 179 | CHPF | chondroitin polymerizing factor [Source:HGNC Symbol;Acc:HGNC:24291] | 7960 | -0.152 | 0.0027 | No |
| 180 | NDOR1 | NADPH dependent diflavin oxidoreductase 1 [Source:HGNC Symbol;Acc:HGNC:29838] | 7982 | -0.154 | 0.0028 | No |
| 181 | FXN | frataxin [Source:HGNC Symbol;Acc:HGNC:3951] | 8057 | -0.160 | -0.0011 | No |
| 182 | ELOVL2 | ELOVL fatty acid elongase 2 [Source:HGNC Symbol;Acc:HGNC:14416] | 8080 | -0.161 | -0.0009 | No |
| 183 | MTRR | 5-methyltetrahydrofolate-homocysteine methyltransferase reductase [Source:HGNC Symbol;Acc:HGNC:7473] | 8087 | -0.162 | 0.0006 | No |
| 184 | AGXT | alanine--glyoxylate and serine--pyruvate aminotransferase [Source:HGNC Symbol;Acc:HGNC:341] | 8115 | -0.164 | 0.0005 | No |
| 185 | SLC1A1 | solute carrier family 1 member 1 [Source:HGNC Symbol;Acc:HGNC:10939] | 8157 | -0.167 | -0.0008 | No |
| 186 | ACSL3 | acyl-CoA synthetase long chain family member 3 [Source:HGNC Symbol;Acc:HGNC:3570] | 8170 | -0.169 | 0.0003 | No |
| 187 | ACLY | ATP citrate lyase [Source:HGNC Symbol;Acc:HGNC:115] | 8173 | -0.169 | 0.0022 | No |
| 188 | GCDH | glutaryl-CoA dehydrogenase [Source:HGNC Symbol;Acc:HGNC:4189] | 8180 | -0.170 | 0.0038 | No |
| 189 | HYAL4 | hyaluronidase 4 [Source:HGNC Symbol;Acc:HGNC:5323] | 8209 | -0.172 | 0.0037 | No |
| 190 | ACSM1 | acyl-CoA synthetase medium chain family member 1 [Source:HGNC Symbol;Acc:HGNC:18049] | 8210 | -0.172 | 0.0057 | No |
| 191 | BLMH | bleomycin hydrolase [Source:HGNC Symbol;Acc:HGNC:1059] | 8219 | -0.173 | 0.0072 | No |
| 192 | CHST15 | carbohydrate sulfotransferase 15 [Source:HGNC Symbol;Acc:HGNC:18137] | 8220 | -0.173 | 0.0093 | No |
| 193 | LPO | lactoperoxidase [Source:HGNC Symbol;Acc:HGNC:6678] | 8298 | -0.178 | 0.0053 | No |
| 194 | B3GNT3 | "UDP-GlcNAc:betaGal beta-1,3-N-acetylglucosaminyltransferase 3 [Source:HGNC Symbol;Acc:HGNC:13528]" | 8361 | -0.183 | 0.0026 | No |
| 195 | XDH | xanthine dehydrogenase [Source:HGNC Symbol;Acc:HGNC:12805] | 8366 | -0.183 | 0.0045 | No |
| 196 | ELOVL1 | ELOVL fatty acid elongase 1 [Source:HGNC Symbol;Acc:HGNC:14418] | 8406 | -0.186 | 0.0037 | No |
| 197 | MPST | mercaptopyruvate sulfurtransferase [Source:HGNC Symbol;Acc:HGNC:7223] | 8457 | -0.190 | 0.0020 | No |
| 198 | OGDH | oxoglutarate dehydrogenase [Source:HGNC Symbol;Acc:HGNC:8124] | 8503 | -0.194 | 0.0008 | No |
| 199 | CHST8 | carbohydrate sulfotransferase 8 [Source:HGNC Symbol;Acc:HGNC:15993] | 8526 | -0.196 | 0.0015 | No |
| 200 | GCLC | glutamate-cysteine ligase catalytic subunit [Source:HGNC Symbol;Acc:HGNC:4311] | 8534 | -0.196 | 0.0033 | No |
| 201 | SLC25A10 | solute carrier family 25 member 10 [Source:HGNC Symbol;Acc:HGNC:10980] | 8552 | -0.198 | 0.0043 | No |
| 202 | SLC35D2 | solute carrier family 35 member D2 [Source:HGNC Symbol;Acc:HGNC:20799] | 8714 | -0.210 | -0.0059 | No |
| 203 | MAT1A | methionine adenosyltransferase 1A [Source:HGNC Symbol;Acc:HGNC:6903] | 8753 | -0.213 | -0.0063 | No |
| 204 | SMS | spermine synthase [Source:HGNC Symbol;Acc:HGNC:11123] | 8884 | -0.223 | -0.0139 | No |
| 205 | GSTT4 | glutathione S-transferase theta 4 [Source:HGNC Symbol;Acc:HGNC:26930] | 8923 | -0.226 | -0.0142 | No |
| 206 | ABCD1 | ATP binding cassette subfamily D member 1 [Source:HGNC Symbol;Acc:HGNC:61] | 8930 | -0.226 | -0.0119 | No |
| 207 | SULT2A1 | sulfotransferase family 2A member 1 [Source:HGNC Symbol;Acc:HGNC:11458] | 8934 | -0.227 | -0.0094 | No |
| 208 | PDHA2 | pyruvate dehydrogenase E1 subunit alpha 2 [Source:HGNC Symbol;Acc:HGNC:8807] | 9124 | -0.241 | -0.0215 | No |
| 209 | ACSL5 | acyl-CoA synthetase long chain family member 5 [Source:HGNC Symbol;Acc:HGNC:16526] | 9136 | -0.243 | -0.0194 | No |
| 210 | HSPA9 | heat shock protein family A (Hsp70) member 9 [Source:HGNC Symbol;Acc:HGNC:5244] | 9173 | -0.246 | -0.0193 | No |
| 211 | ACACA | acetyl-CoA carboxylase alpha [Source:HGNC Symbol;Acc:HGNC:84] | 9177 | -0.246 | -0.0165 | No |
| 212 | GNMT | glycine N-methyltransferase [Source:HGNC Symbol;Acc:HGNC:4415] | 9208 | -0.248 | -0.0159 | No |
| 213 | MTR | 5-methyltetrahydrofolate-homocysteine methyltransferase [Source:HGNC Symbol;Acc:HGNC:7468] | 9213 | -0.249 | -0.0132 | No |
| 214 | ACSF2 | acyl-CoA synthetase family member 2 [Source:HGNC Symbol;Acc:HGNC:26101] | 9219 | -0.250 | -0.0106 | No |
| 215 | PHGDH | phosphoglycerate dehydrogenase [Source:HGNC Symbol;Acc:HGNC:8923] | 9245 | -0.252 | -0.0095 | No |
| 216 | GNS | glucosamine (N-acetyl)-6-sulfatase [Source:HGNC Symbol;Acc:HGNC:4422] | 9345 | -0.259 | -0.0142 | No |
| 217 | DPEP1 | dipeptidase 1 [Source:HGNC Symbol;Acc:HGNC:3002] | 9353 | -0.260 | -0.0116 | No |
| 218 | GGT5 | gamma-glutamyltransferase 5 [Source:HGNC Symbol;Acc:HGNC:4260] | 9361 | -0.261 | -0.0090 | No |
| 219 | CLIC5 | chloride intracellular channel 5 [Source:HGNC Symbol;Acc:HGNC:13517] | 9382 | -0.262 | -0.0075 | No |
| 220 | B3GNT4 | "UDP-GlcNAc:betaGal beta-1,3-N-acetylglucosaminyltransferase 4 [Source:HGNC Symbol;Acc:HGNC:15683]" | 9500 | -0.274 | -0.0134 | No |
| 221 | SLC26A2 | solute carrier family 26 member 2 [Source:HGNC Symbol;Acc:HGNC:10994] | 9523 | -0.276 | -0.0118 | No |
| 222 | SULT2B1 | sulfotransferase family 2B member 1 [Source:HGNC Symbol;Acc:HGNC:11459] | 9696 | -0.291 | -0.0219 | No |
| 223 | CHST5 | carbohydrate sulfotransferase 5 [Source:HGNC Symbol;Acc:HGNC:1973] | 9732 | -0.295 | -0.0211 | No |
| 224 | CSGALNACT2 | chondroitin sulfate N-acetylgalactosaminyltransferase 2 [Source:HGNC Symbol;Acc:HGNC:24292] | 9773 | -0.298 | -0.0207 | No |
| 225 | SULT1C2 | sulfotransferase family 1C member 2 [Source:HGNC Symbol;Acc:HGNC:11456] | 9843 | -0.305 | -0.0224 | No |
| 226 | CHAC1 | ChaC glutathione specific gamma-glutamylcyclotransferase 1 [Source:HGNC Symbol;Acc:HGNC:28680] | 9853 | -0.307 | -0.0194 | No |
| 227 | NAT8 | N-acetyltransferase 8 (putative) [Source:HGNC Symbol;Acc:HGNC:18069] | 9879 | -0.310 | -0.0177 | No |
| 228 | CHST4 | carbohydrate sulfotransferase 4 [Source:HGNC Symbol;Acc:HGNC:1972] | 9941 | -0.315 | -0.0187 | No |
| 229 | PPT2 | palmitoyl-protein thioesterase 2 [Source:HGNC Symbol;Acc:HGNC:9326] | 10103 | -0.331 | -0.0274 | No |
| 230 | SLC19A3 | solute carrier family 19 member 3 [Source:HGNC Symbol;Acc:HGNC:16266] | 10126 | -0.334 | -0.0251 | No |
| 231 | MTHFD1 | "methylenetetrahydrofolate dehydrogenase, cyclohydrolase and formyltetrahydrofolate synthetase 1 [Source:HGNC Symbol;Acc:HGNC:7432]" | 10249 | -0.346 | -0.0306 | No |
| 232 | UGDH | UDP-glucose 6-dehydrogenase [Source:HGNC Symbol;Acc:HGNC:12525] | 10279 | -0.349 | -0.0287 | No |
| 233 | AMD1 | adenosylmethionine decarboxylase 1 [Source:HGNC Symbol;Acc:HGNC:457] | 10415 | -0.363 | -0.0350 | No |
| 234 | GCLM | glutamate-cysteine ligase modifier subunit [Source:HGNC Symbol;Acc:HGNC:4312] | 10439 | -0.366 | -0.0324 | No |
| 235 | BAAT | bile acid-CoA:amino acid N-acyltransferase [Source:HGNC Symbol;Acc:HGNC:932] | 10523 | -0.376 | -0.0344 | No |
| 236 | GDAP1L1 | ganglioside induced differentiation associated protein 1 like 1 [Source:HGNC Symbol;Acc:HGNC:4213] | 10607 | -0.384 | -0.0363 | No |
| 237 | DLST | dihydrolipoamide S-succinyltransferase [Source:HGNC Symbol;Acc:HGNC:2911] | 10634 | -0.386 | -0.0337 | No |
| 238 | ST3GAL4 | "ST3 beta-galactoside alpha-2,3-sialyltransferase 4 [Source:HGNC Symbol;Acc:HGNC:10864]" | 10688 | -0.391 | -0.0332 | No |
| 239 | ACSL1 | acyl-CoA synthetase long chain family member 1 [Source:HGNC Symbol;Acc:HGNC:3569] | 10705 | -0.394 | -0.0297 | No |
| 240 | HMGCS1 | 3-hydroxy-3-methylglutaryl-CoA synthase 1 [Source:HGNC Symbol;Acc:HGNC:5007] | 10829 | -0.408 | -0.0345 | No |
| 241 | MVK | mevalonate kinase [Source:HGNC Symbol;Acc:HGNC:7530] | 10867 | -0.412 | -0.0324 | No |
| 242 | SULT1B1 | sulfotransferase family 1B member 1 [Source:HGNC Symbol;Acc:HGNC:17845] | 10893 | -0.416 | -0.0294 | No |
| 243 | MVD | mevalonate diphosphate decarboxylase [Source:HGNC Symbol;Acc:HGNC:7529] | 10934 | -0.423 | -0.0274 | No |
| 244 | ACACB | acetyl-CoA carboxylase beta [Source:HGNC Symbol;Acc:HGNC:85] | 10955 | -0.425 | -0.0239 | No |
| 245 | CTH | cystathionine gamma-lyase [Source:HGNC Symbol;Acc:HGNC:2501] | 11108 | -0.444 | -0.0305 | No |
| 246 | NDST1 | N-deacetylase and N-sulfotransferase 1 [Source:HGNC Symbol;Acc:HGNC:7680] | 11241 | -0.462 | -0.0354 | No |
| 247 | ELOVL5 | ELOVL fatty acid elongase 5 [Source:HGNC Symbol;Acc:HGNC:21308] | 11272 | -0.467 | -0.0321 | No |
| 248 | ACSL4 | acyl-CoA synthetase long chain family member 4 [Source:HGNC Symbol;Acc:HGNC:3571] | 11327 | -0.475 | -0.0306 | No |
| 249 | METTL16 | "methyltransferase 16, N6-methyladenosine [Source:HGNC Symbol;Acc:HGNC:28484]" | 11346 | -0.478 | -0.0263 | No |
| 250 | PDK4 | pyruvate dehydrogenase kinase 4 [Source:HGNC Symbol;Acc:HGNC:8812] | 11384 | -0.483 | -0.0234 | No |
| 251 | DCN | decorin [Source:HGNC Symbol;Acc:HGNC:2705] | 11402 | -0.487 | -0.0188 | No |
| 252 | GSTA1 | glutathione S-transferase alpha 1 [Source:HGNC Symbol;Acc:HGNC:4626] | 11608 | -0.517 | -0.0288 | No |
| 253 | CHSY1 | chondroitin sulfate synthase 1 [Source:HGNC Symbol;Acc:HGNC:17198] | 11628 | -0.521 | -0.0240 | No |
| 254 | CSAD | cysteine sulfinic acid decarboxylase [Source:HGNC Symbol;Acc:HGNC:18966] | 11649 | -0.525 | -0.0192 | No |
| 255 | ST3GAL2 | "ST3 beta-galactoside alpha-2,3-sialyltransferase 2 [Source:HGNC Symbol;Acc:HGNC:10863]" | 11721 | -0.536 | -0.0184 | No |
| 256 | EEF1G | eukaryotic translation elongation factor 1 gamma [Source:HGNC Symbol;Acc:HGNC:3213] | 11767 | -0.544 | -0.0154 | No |
| 257 | CHST1 | carbohydrate sulfotransferase 1 [Source:HGNC Symbol;Acc:HGNC:1969] | 12049 | -0.606 | -0.0303 | No |
| 258 | EDNRB | endothelin receptor type B [Source:HGNC Symbol;Acc:HGNC:3180] | 12075 | -0.613 | -0.0248 | No |
| 259 | ST3GAL1 | "ST3 beta-galactoside alpha-2,3-sialyltransferase 1 [Source:HGNC Symbol;Acc:HGNC:10862]" | 12092 | -0.619 | -0.0186 | No |
| 260 | DIP2A | disco interacting protein 2 homolog A [Source:HGNC Symbol;Acc:HGNC:17217] | 12433 | -0.719 | -0.0368 | No |
| 261 | MICAL1 | "microtubule associated monooxygenase, calponin and LIM domain containing 1 [Source:HGNC Symbol;Acc:HGNC:20619]" | 12483 | -0.742 | -0.0318 | No |
| 262 | SP1 | Sp1 transcription factor [Source:HGNC Symbol;Acc:HGNC:11205] | 12557 | -0.777 | -0.0281 | No |
| 263 | SLC19A2 | solute carrier family 19 member 2 [Source:HGNC Symbol;Acc:HGNC:10938] | 12600 | -0.798 | -0.0218 | No |
| 264 | SOD2 | superoxide dismutase 2 [Source:HGNC Symbol;Acc:HGNC:11180] | 12685 | -0.853 | -0.0181 | No |
| 265 | CIAO1 | cytosolic iron-sulfur assembly component 1 [Source:HGNC Symbol;Acc:HGNC:14280] | 12687 | -0.854 | -0.0079 | No |
| 266 | AASS | aminoadipate-semialdehyde synthase [Source:HGNC Symbol;Acc:HGNC:17366] | 12799 | -0.968 | -0.0050 | No |
| 267 | MAT2A | methionine adenosyltransferase 2A [Source:HGNC Symbol;Acc:HGNC:6904] | 12852 | -1.066 | 0.0038 | No |
Table: GSEA details [plain text format]

  

Fig 2: GOBP\_SULFUR\_COMPOUND\_METABOLIC\_PROCESS      
 Blue-Pink O' Gram in the Space of the Analyzed GeneSet

  

Fig 3: GOBP\_SULFUR\_COMPOUND\_METABOLIC\_PROCESS: Random ES distribution      
 Gene set null distribution of ES for **GOBP\_SULFUR\_COMPOUND\_METABOLIC\_PROCESS**
